# Association between germline homeobox B13 (HOXB13) G84E allele and prostate cancer susceptibility: a meta-analysis and trial sequential analysis

**DOI:** 10.18632/oncotarget.11937

**Published:** 2016-09-10

**Authors:** Jianzhong Zhang, Li Xiao, Zhiqiang Qin, Aiming Xu, Kai Zhao, Chao Liang, Chenkui Miao, Jundong Zhu, Wei Chen, Yibo Hua, Yiyang Liu, Chao Zhang, Yajie Yu, Shifeng Su, Zengjun Wang

**Affiliations:** ^1^ State Key Laboratory of Reproductive Medicine and Department of Urology, The First Affiliated Hospital of Nanjing Medical University, Nanjing, China; ^2^ Department of Urology, The Affiliated Cancer Hospital of Jiangsu Province of Nanjing Medical University, Nanjing, China

**Keywords:** HOXB13, G84E, gene polymorphism, prostate cancer, meta-analysis

## Abstract

Germline HOXB13 G84E mutation (rs138213197) has been described associated with prostate cancer (PCa) susceptibility but results of different studies are inconsistent. We conducted this meta-analysis to evaluate the specific role of this mutation. Relevant available studies were identified by searching the databases Pubmed, Embase and Web of Science. Odds ratios (ORs) and 95% confidence intervals (CIs) were calculated to measure the strength of the association. Subgroup analysis were performed to evaluate the specific role of rs138213197 in disease aggressiveness, diagnostic age and family history. Furthermore, trial sequential analysis (TSA) was conducted for the first time to estimate whether the evidence of the results is sufficient. Our results indicated that significant increased PCa susceptibility was associated with rs138213197 compared with non-carriers (OR = 3.38, 95% CI: 2.45–4.66). Besides, in subgroup analysis, HOXB13 G84E variant was obviously associated with early onset (OR = 2.90, 95% CI: 2.24–3.75), affected relatives (OR = 2.60, 95% CI 2.19–3.10) and highly aggressive disease (OR = 2.38, 95% CI 1.84–3.08). By TSA, the findings in the current study were based on sufficient evidence. Therefore, our results indicated that the G84E mutation in HOXB13 gene might increase susceptibility to PCa.

## INTRODUCTION

Prostate cancer (PCa), as the most common cancer among men, is the second leading cause of tumor death in men of USA [1, 2]. Various risk factors have been reported associated with PCa susceptibility, including age, dietary habits, blood testosterone, infectious disease as well as genetic factors [3–7]. Among these, genetic factors might play an important role, which can explain 16% to 45% of the risk of PCa [8, 9]. Singe nucleotide polymorphism (SNP), as an important genetic factor, has been widely studied in recent years [10].

Germline Homeobox B13 (HOXB13) encodes a transcription factor which belongs to the highly conserved homeobox gene family. Many studies have demonstrated the role of HOXB13 in embryonic development and tumor suppression [11, 12]. In PCa, HOXB13 gene expression can suppress androgen receptor signaling and was considered to be a PCa suppressor [13]. In 2012, a novel rare G84E allele in HOXB13 (rs138213197) was discovered to contribute to higher PCa risk. The mutation was identified by a substitution of adenine for guanine in the second position of codon 84, thus leading to the replacement of glutamic acid for glycine. Mutation in the conserved domain might result in HOXB13 protein binding to members of the MEIS protein family and interfere androgen receptor signaling [14]. Since then, increasing number of literature have researched the relationship between rs138213197 and PCa risk [15–29].

In most studies, rs138213197 has been revealed to be a significant risk factor associated with PCa incidence [14–20, 22–25, 27–29]. Moreover, the presence of rs138213197 in PCa patients usually signified early-onset age and family history. Meanwhile, some studies have presented contradictory or insignificant results. For instance, Laitinen et al. found no significant relationship between rs138213197 and PCa risk [21]. Albitar et al. found lower rs138213197 mutation in PCa patients through studying 232 PCa patients and 110 controls [26]. However, the results might own to limited sample size or other potential bias. Besides, lack of further research in different stratified analysis prevented comprehensive understanding of the relationship in some recent meta-analyses [30, 31]. Here, we conducted such meta-analysis by including all eligible literature in order to get a more precise conclusion.

## RESULTS

### Summary of the enrolled studies

All remaining articles were checked to prevent overlapping datasets (Figure 2). A total of 16 case-control studies including 50,048 cases and 123,211 controls were involved in the current meta-analysis at last (Table 1). Of the 16 enrolled studies, there were 12 studies of Caucasian and 4 mixed ethnicity studies (Caucasian occupy the majority). In regard to the control source, the studies could be divided into population-based (PB) control, hospital-based (HB) control, and family-based (FB) control. Four genotyping methods were applied in enrolled studies: TaqMan assay, Sanger sequencing, MassARRAY iPLEX, and Illumina SNP.

**Figure 1:**
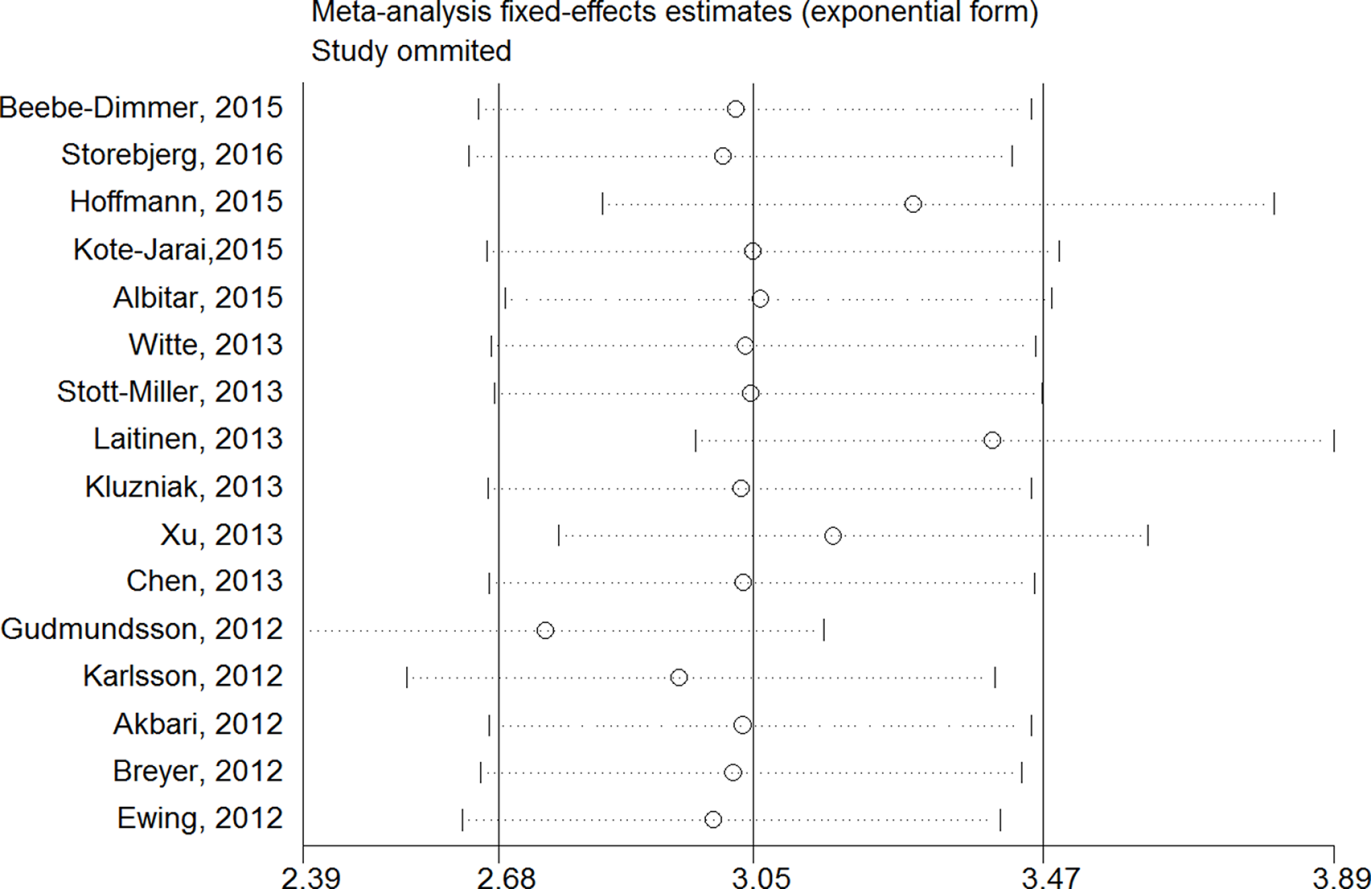
Sensitivity analysis of each included study in this meta-analysis by omitting each data set in the meta-analysis

**Figure 2:**
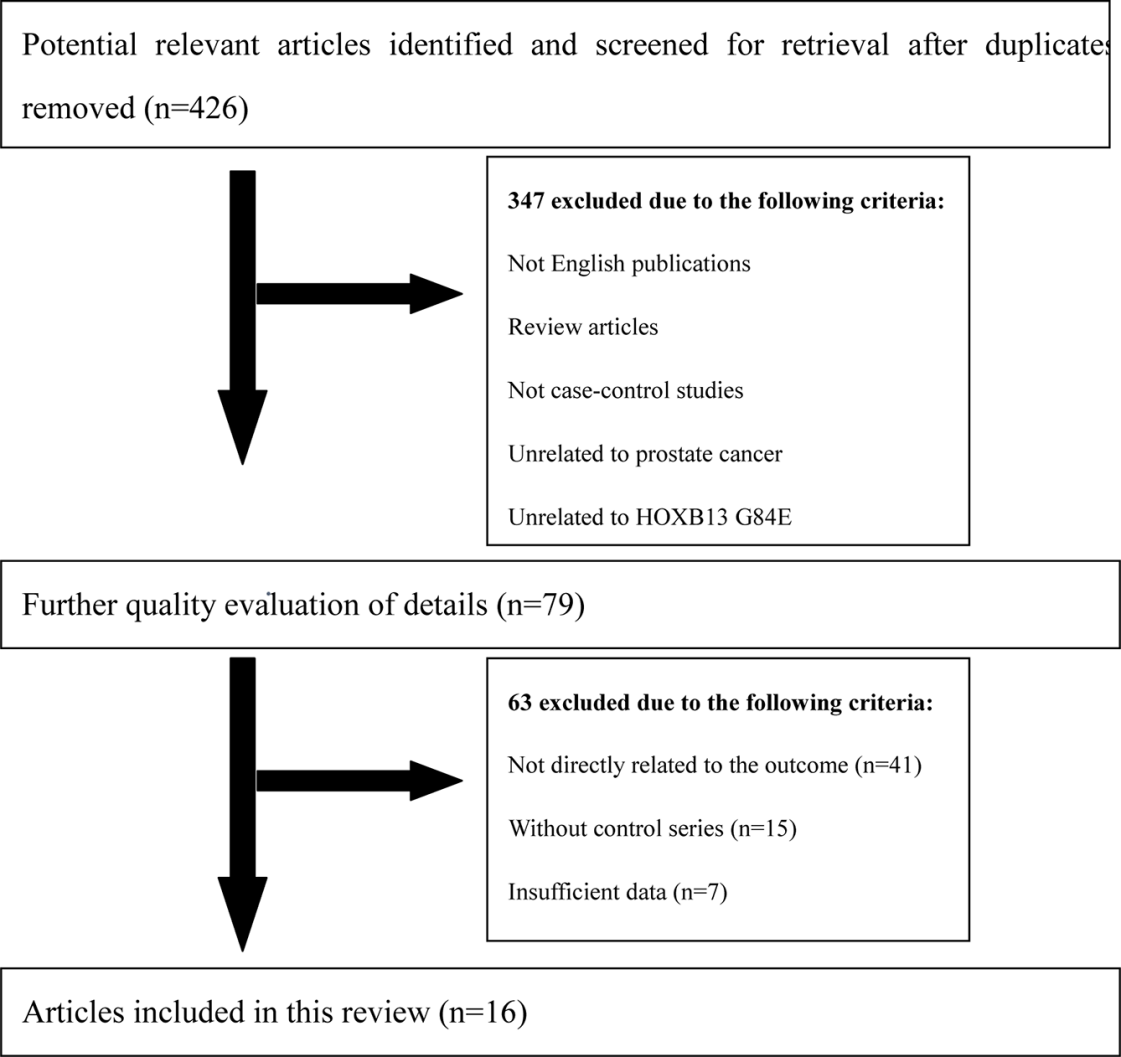
Flow diagram of literature search and selection process

**Table 1:** Main characteristics of trials included in this meta-analysis

First author, publication year	Study design	Ethnicity	Genotyping methods	Mutation		Wildtype	
				Patients	Controls	Patients	Controls
Beebe-Dimmer, 2015	HB	Caucasian	TaqMan Assay	19	23	1343	5875
Storebjerg, 2016	HB	Caucasian	Sanger sequencing	25	8	970	1614
Hoffmann, 2015	PB	Caucasian	Sanger sequencing	74	230	3902	29287
Kote-Jarai,2015	HB	Caucasian	TaqMan Assay	134	27	8519	5224
Albitar, 2015	HB	Caucasian	Sanger sequencing	2	1	230	109
Witte, 2013	HB/FB	Mixed	TaqMan Assay	20	3	1625	1016
Stott-Miller, 2013	PB	Caucasian	TaqMan Assay	17	5	1293	1254
Laitinen, 2013	PB/HB	Caucasian	TaqMan Assay	160	28	4411	895
Kluzniak, 2013	PB	Caucasian	TaqMan Assay	20	3	3495	2601
Xu, 2013	FB	Caucasian	MassARRAY iPLEX	154	36	172	81
Chen, 2013	HB	Mixed	MassARRAY iPLEX	7	6	701	2485
Gudmundsson, 2012	HB	Caucasian	Illumina SNP chips	55	50	9933	61944
Karlsson, 2012	PB/HB	Caucasian	MassARRAY iPLEX	221	61	4682	4528
Akbari, 2012	HB	Mixed	Sanger sequencing	10	2	1843	2223
Breyer, 2012	HB	Mixed	TaqMan Assay	20	2	908	928
Ewing, 2012	HB	Caucasian	TaqMan Assay	72	4	5011	2658

### Sensitivity analysis

Sensitivity analysis was utilized to detect the influence of each study on the pooled OR by repeating the meta-analysis while omitting one single study each time. Our sensitivity analysis did not indicate alterations in the results if any individual study was excluded (Figure 1).

### Quantitative synthesis results

**Figure 3:**
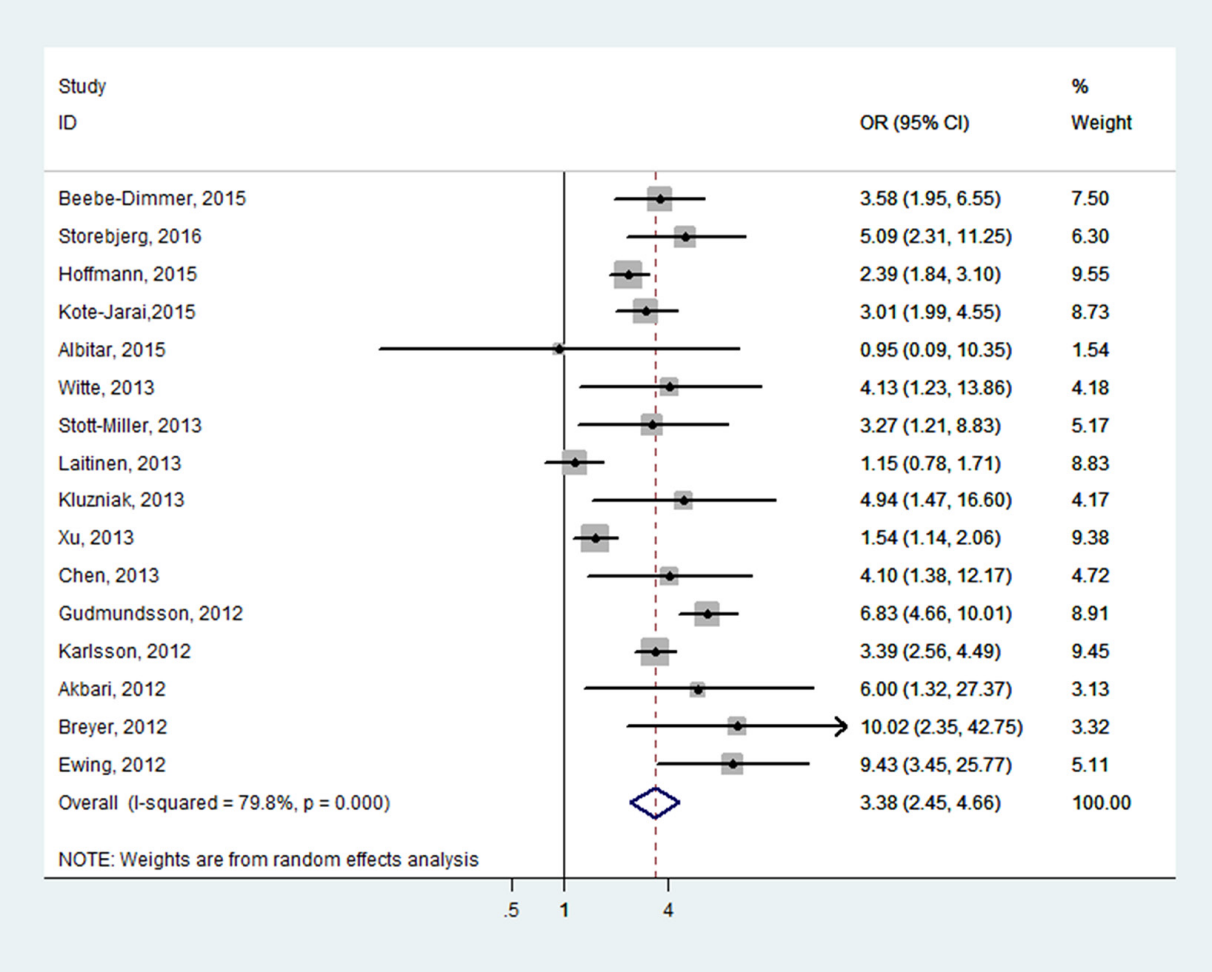
Forest plots of the association between HOXB13 G84E and prostate cancer susceptibility

**Figure 4:**
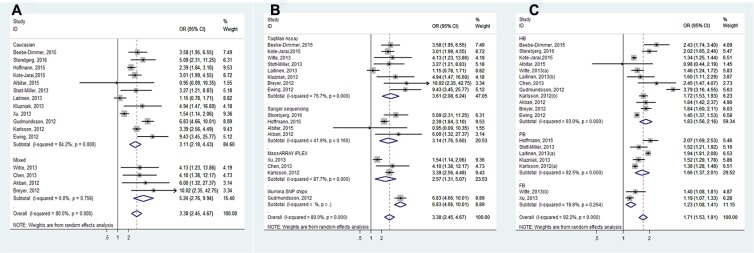
Forest plots of subgroup analysis of the association between HOXB13 G84E allele and prostate cancer susceptibility. (**A**) stratified by ethnicity; (**B**) stratified by genotyping method; (**C**) stratified by source of control

Overall, the pooled OR of the enrolled 16 studies was 3.38 (95% CI: 2.45–4.66) in a random-effect model, which indicated a strong association between HOXB13 G84E mutation and PCa susceptibility (Figure 3). In the subgroup analysis by ethnicity, the results were positive both in Caucasian populations and mixed populations. (Caucasian populations: pooled OR = 3.11, 95% CI: 2.18–4.43; Mixed populations: pooled OR = 5.24, 95% CI: 2.76–9.94) (Figure 4A). Furthermore, when the studies were stratified by genotyping method, the results were detected significant in all subgroups (TaqMan assay: pooled OR = 3.61, 95% CI: 2.08–6.24; Sanger sequencing: pooled OR = 3.14, 95% CI: 1.76–5.60, MassARRAY iPLEX: pooled OR = 2.57, 95% CI: 1.31–5.07; Illumina SNP: pooled OR = 3.38, 95% CI: 2.45–4.67) (Figure 4B). In addition, in the stratified analysis by source of control, the significant results were also detected in all the subgroups (hospital based controls: OR = 1.83, 95% CI: 1.56–2.16; population based controls OR = 1.66, 95% CI: 1.37–2.01; family based controls: OR = 1.71, 95% CI: 1.53–1.91) (Figure 4C).

**Figure 5:**
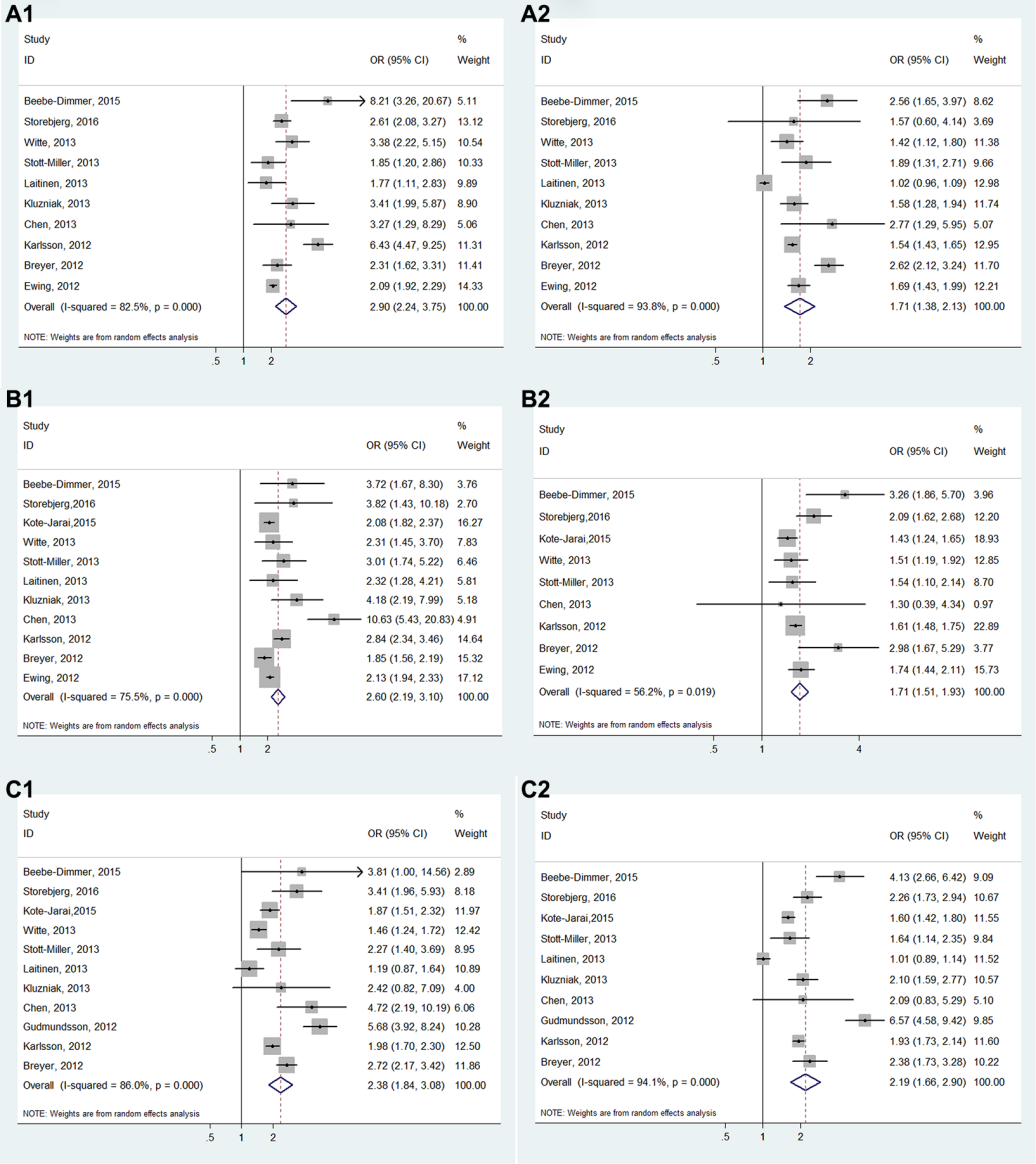
Forest plots of subgroup analysis of the association between HOXB13 G84E allele and prostate cancer susceptibility (**A**) stratified by age (A1: early-onset; A2: late-onset); (**B**) stratified by family history (B1: with relatives affected; B2: without relatives affected); (**C**) stratified by disease aggressive (C1: more aggressive disease; C2: less aggressive disease).

**Figure 6:**
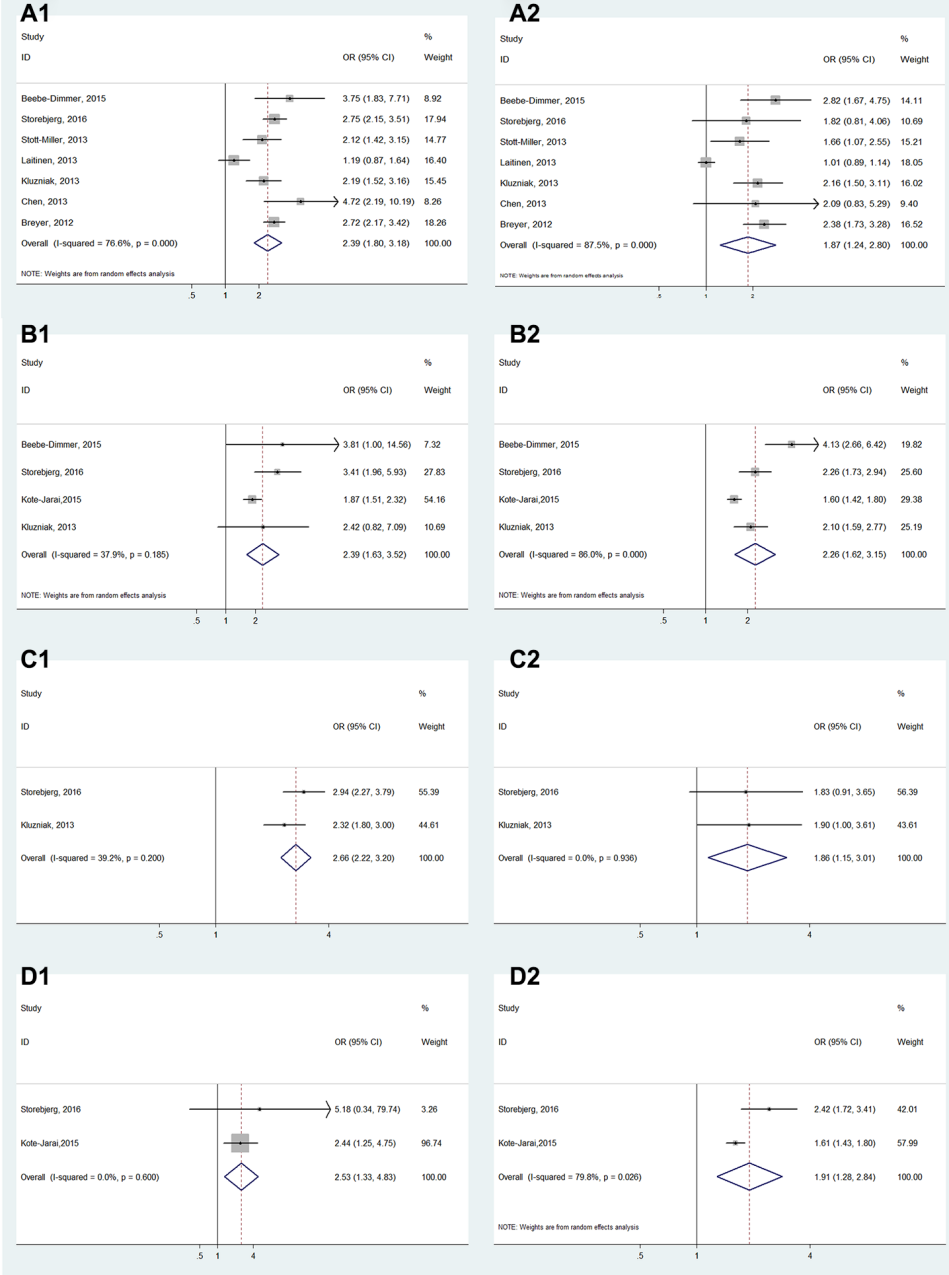
Forest plots of subgroup analysis of the association between HOXB13 G84E allele and prostate cancer susceptibility (**A**) stratified by cancer grading (A1: Gleason score ≥ 7; A2: Gleason < 7); (**B**) stratified by staging (B1: tumor stage ≥ T3; B2: tumor stage ≤ T2); (**C**) stratified by blood PSA level (C1: PSA ≥ 10 ng/mL; C2: PSA < 10 ng/mL) and (**D**) stratified by lymph node metastasis (D1: tumor stage > N0; D2: tumor stage = N0).

For subgroup analyses of the age, family history and aggressive, all results showed a significant association between HOXB13 G84E mutation and PCa susceptibility (Early-onset: OR = 2.09, 95% CI: 2.24–3.75; Late-onset: OR = 1.71, 95% CI: 1.38–2.13; with family history: OR = 2.60, 95% CI: 2.19–3.10; without family history: OR = 1.71, 95% CI: 1.51–1.93; More-aggressive: OR = 2.38, 95% CI: 1.84–3.08; Less-aggressive: OR = 2.19, 95% CI: 1.66–2.90) (Figure 5). In addition, the results also all showed a significant association in the subgroup analyses of PCa grading, staging, blood PSA level and lymph node metastasis (Gleason score ≥ 7: OR = 2.39, 95% CI: 1.80–3.18; Gleason score < 7: OR = 1.87, 95% CI: 1.24–2.80; Postoperative T stage ≥ T3: OR = 2.39, 95% CI: 1.63–3.52; Postoperative T stage ≤ T2: OR = 2.26, 95% CI: 1.62–3.15; PSA at diagnosis ≥ 10 ng/mL: OR = 2.66, 95% CI: 2.22–3.20; PSA at diagnosis < 10 ng/mL: OR = 1.86, 95% CI: 1.15–3.01; Postoperative N stage > pN0: OR = 2.53, 95% CI: 1.33–4.83; Postoperative N stage = pN0: OR = 1.91, 95% CI: 1.28–2.84) (Figure 6). (The Gleason scores were derived from biopsies in three studies [21, 23, 25] and prostatectomies in two articles [15, 27] and mixed in one research [20], The T-stages were identified clinically in one study [23] and pathologically in two articles [22, 27].)

### Publication bias

**Figure 7:**
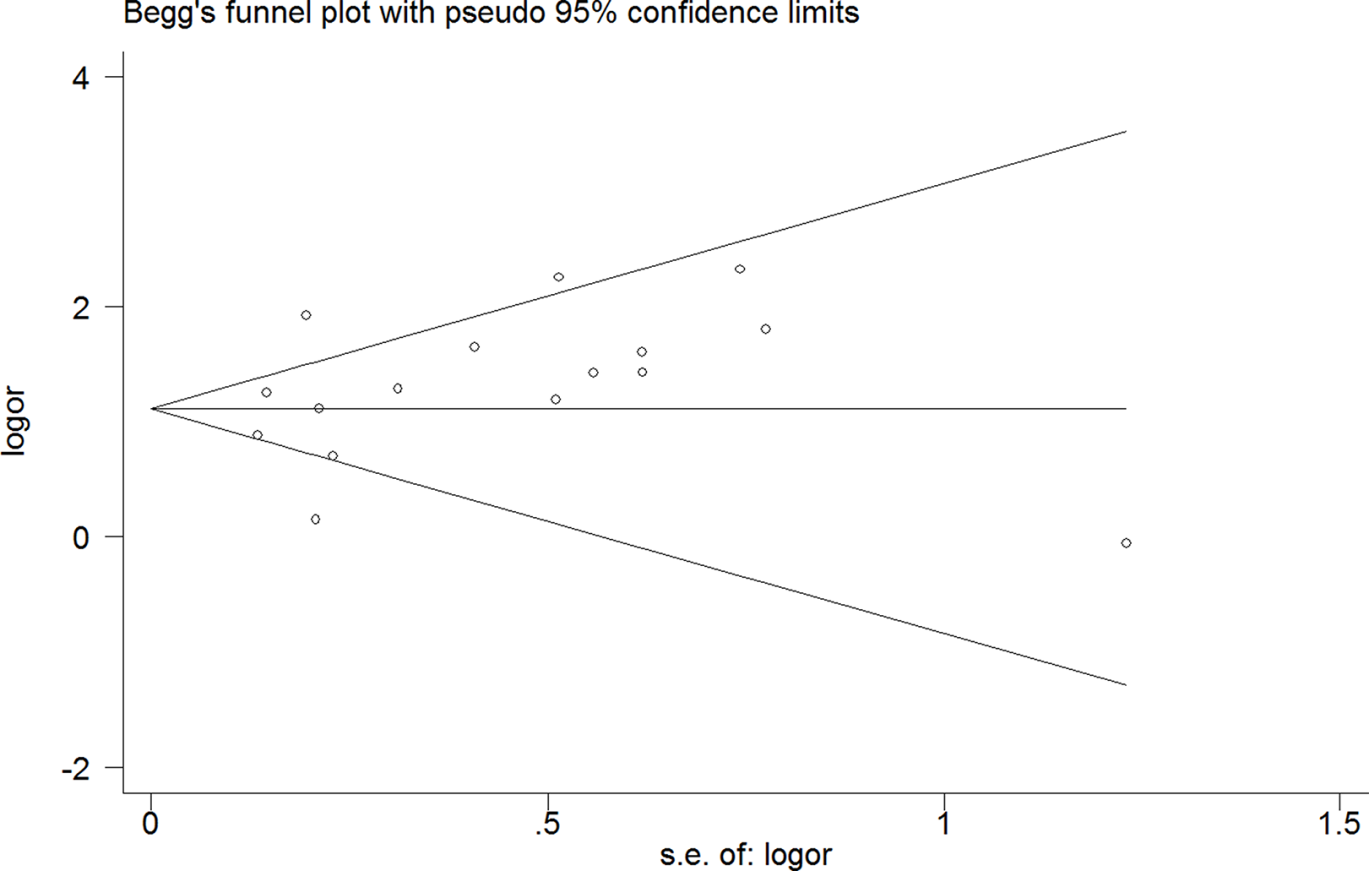
Funnel plot of the association between HOXB13 G84E allele and prostate cancer susceptibility

Begg's tests were utilized to detect if there existed potential publication bias. The funnel plot indicated no evidence of obvious asymmetrical (*P* = 0.418) (Figure 7).

### Trial sequential analysis results

**Figure 8:**
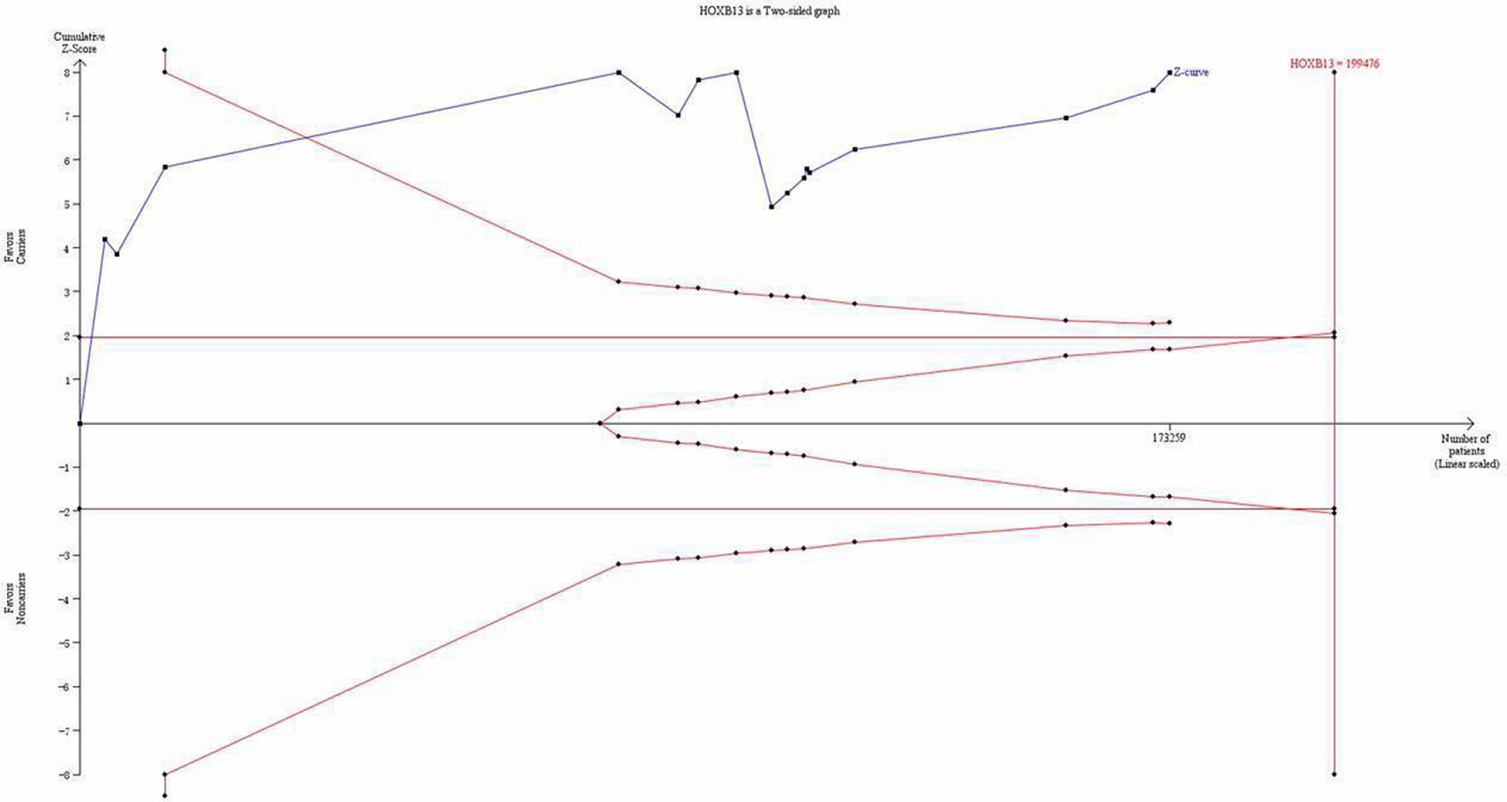
Trial sequential analysis of the association between between HOXB13 G84E allele and the risk of prostate cancer The required information size was calculated based on a two side α = 5%, β = 10% (power 90%), and a relative risk reduction of 10%.

For the first time, trial sequential analysis (TSA) was conducted for a more comprehensive assessment in our current meta-analysis. Although the number of the cases and controls have not achieved the required information size, the cumulative Z-curve has crossed the monitoring boundaries already (Figure 8), demonstrating that our results were based on sufficient evidence.

## DISCUSSION

Mutations in key tumor suppressor genes, oncogenes, or mismatch repair genes might play a vital role in tumor occurrence and development [32]. Recently, a rare but recurrent mutation HOXB13 G84E was identified to be associated with a significant increase risk of familial PCa [14]. This initial discovery of HOXB13 G84E mutation was based on screening more than 200 genes in the 17q21–22 region by sequencing Germline DNA from 94 youngest men with familial PCa. Carrier frequency of this allele was subsequently tested in a unique Caucasian sample of 5,011 cases and in 1,401 controls and a 20.1-fold increased PCa risk among the rare allele carriers was revealed [14]. A number of subsequent studies have investigated the association between HOXB13 G84E allele and PCa susceptibility. Nevertheless, the outcome remains inconsistent. Though most studies revealed that HOXB13 G84E mutation was associated with an increased risk of PCa in total and stratified analysis, some researchers hold different opinions [21, 26]. The conflicting results in their study might partially be aroused from different distributions of the included samples, the relatively small sample size and the possible effect of the mutation on PCa risk.

Meta-analyses have a greater power than a single study by combining all eligible studies. In the current study, there existed several advantages: (1) The sample size is larger than any single study, making our results more reliable and precise. (2) The Begg's test and sensitivity analysis showed no publication bias and low-quality study. (3) Subgroup analyses were conducted to explore the association between HOXB13 G84E allele and PCa susceptibility in different ethnicities, source of controls, genotyping methods, tumor stages, blood PSA levels, and Gleason scores. (4) Our results were based on sufficient evidence, which was proved by TSA for the first time.

In the present meta-analysis, the results showed the mutation is associated with a 3.38-fold increased risk of PCa in total. Moreover, subgroup analyses in different ethnicities, source of controls and genotyping methods were conducted. Results of these subgroup analyses indicated significant association between rs138213197 and PCa risk. Noticeably, subgroup analyses were also performed according to disease aggressive and all the results indicated significant association, patients with more aggressive disease are more likely to carry the mutation. In conclusion, the present results indicated that the G84E mutation in HOXB13 gene might increase susceptibility to PCa.

TSA is an approach that combines an a required information size with the adaptation of monitoring boundaries to evaluate the accumulating data [33]. In our meta-analysis, although the number of the cases and controls have not achieved the required information size, the cumulative Z-curve has crossed the monitoring boundaries already, indicating our meta-analysis was based on firm evidence of effect.

However, some limitations should also be emphasized. (1) Most populations included in this meta-analysis were Caucasian ethnicity, and more populations from other ethnicities will be required in future research. (2) The number of included studies in some subgroups was relatively small, with limited statistical power to investigate the real association. (3) Adjusted estimates could not be performed in our analysis without enough data for the adjustment by other PCa covariates such as age, life-style and so on. (4) Though TSA was conducted for the first time in this meta-analysis to assess the risk of random error, the shortage of TSA itself could not be avoided. More studies by standardized unbiased methods are required, which can offer more detailed individual data of high quality. (5) In our meta-analysis, the source of Gleason score and T-stage were different in various included studies, which might induce potential inaccuracy.

## MATERIALS AND METHODS

This meta-analysis was strictly performed according to the preferred reporting items of the systematic reviews and meta-analysis (PRISMA) statement [34].

### Search strategy

Online databases including Pubmed, Embase and Web of Science were searched to identify relevant literature published until 20 April 2016. The combination of the following search items were utilized: “Germline Homeobox B13” or “HOXB13”, “polymorphism” or “rs138213197” or “G84E” or “p.Gly84Glu”, and “Prostate”. Additional eligible studies were searched manually from reference of original studies or reviews. Following criteria were utilized to select the published literature: (1) English publications; (2) case-control studies; (3) studies concerning the association between HOXB13 G48E variant and PCa risk.

To maintain the quality of the meta-analysis, studies were excluded when: (1) no clear definitions of the study design, population, country of origin or the outcome assessment; (2) no sufficient data for estimating an odds ratio (OR) with 95% confidence interval (CI); (3) without control series; (4) duplicates of previous publication. Sensitivity analyses were also performed to avoid biases in the results due to certain low-quality studies (Figure 1).

### Data extraction

All eligible studies were identified by two investigators (Zhang JZ and Qin ZQ) independently to determine whether an individual study was eligible for inclusion. The information were recorded in a standardized form and the extracted elements included: first author's name, year of publication, control source [population based (PB) or hospital based (HB)], ethnicity, genotyping method, the number of HOXB13 G84E mutation carriers and non-carriers respectively. All of the aforementioned data are comprehensively detailed in Table 1.

### Statistical analysis

Sensitivity analysis was conducted firstly by calculating the results again by omitting one single study each time. The pooled odds ratios (ORs) with 95% confidence intervals (CIs) were utilized to evaluate the strength of association between rs138213197 and PCa susceptibility. Random-effect pooled ORs and 95% CIs were calculated through the DerSimonian–Laird method and the fixed-effect model based on the Mantel–Haenszel method according to heterogeneity among the pooled studies [35]. Higgins I^2^ statistic and Cochran Q test were applied to assess the heterogeneity [36]. A random-effect model was utilized if significant heterogeneity existed (*p* < 0.05 or I^2^ > 50%); otherwise, the fixed-effect model was applied. An OR value greater than 1 indicated HOXB13 G84E variant was associated with higher PCa susceptibility and was considered statistically significant if the 95% CI did not include 1. After that, subgroup analysis was further carried out by diagnostic age, family history and disease aggressiveness. Early onset was defined as age ≤ 55 years in five papers [14, 17, 19–21], defined as age ≤ 60 years in three studies [15, 23, 28] and defined as age ≤ 65 in two researches [25, 27]. Additionally, patients with family history was defined to have at least one affected first- or second-relatives. At last, highly aggressive PCa was defined as Gleason ≥ 7 and/or T3 or higher and/or node positive and/or metastatic disease. Otherwise, the disease aggressiveness would be considered low. Egger's linear regression test was applied to estimate the publication bias with a funnel plot [37]. *p* < 0.05 was considered statistically significant. The meta-analyses were carried out with Stata12 (StataCorp LP, College Station, TX, USA).

### Trial sequential analysis

Random error can mislead results in meta-analyses. The risk of random error may increase remarkably because of multiple looks on accumulating evidence when new trials emerge [38, 39]. To obtain a more comprehensive assessment of meta-analyses, TSA was conducted in this meta-analysis to control the risk of random error. Sequential monitoring boundaries were utilized to decide whether a trial could be terminated early.

In our study, 5% risk of a type I error and 10% risk of a type II error (a power of 90%) was set in TSA. Furthermore, 10% relative risk increase was predetermined according to the required information size and 90% confidence intervals was provided. The trial sequential analysis software (TSA, version 0.9; Copenhagen Trial Unit, Copenhagen, Denmark, 2011) was utilized in this meta-analysis.

## CONCLUSIONS

Our results indicated that the G84E mutation in HOXB13 gene might increase susceptibility to PCa. Moreover, the effect size of HOXB13 G84E in PCa is more obvious in patients with relatives affected, more aggressive PCa or early-onset PCa.
